# Relationship between circulating tumour DNA and skeletal muscle stores at diagnosis of pancreatic ductal adenocarcinoma: a cross-sectional study

**DOI:** 10.1038/s41598-023-36643-x

**Published:** 2023-06-14

**Authors:** Lauren Hanna, Rav Sellahewa, Catherine E. Huggins, Joanne Lundy, Daniel Croagh

**Affiliations:** 1 Department of Nutrition, Dietetics and Food, School of Clinical Sciences at Monash Health, Monash University, Clayton, VIC Australia; 2 Department of Nutrition and Dietetics, Monash Health, Clayton, VIC Australia; 3 Centre for Innate Immunity and Infectious Diseases, Hudson Institute of Medical Research, Monash University, Clayton, VIC Australia; 4 Department of Surgery, School of Clinical Sciences at Monash Health, Monash University, Clayton, VIC Australia; 5 Department of Upper Gastrointestinal Surgery, Monash Health, Clayton, VIC Australia; 6 Global Obesity Centre (GLOBE), Institute for Health Transformation, School of Health and Social Development, Deakin University, Burwood, VIC Australia; 7 Department of Molecular and Translational Science, Faculty of Medicine, Nursing and Health Sciences, Monash University, Clayton, VIC Australia

**Keywords:** Tumour biomarkers, Pancreatic cancer, Nutrition disorders, Risk factors, Skeletal muscle

## Abstract

Low skeletal muscle index (SMI) and low skeletal muscle radiodensity (SMD) are associated with reduced survival time in pancreatic ductal adenocarcinoma (PDAC). The negative prognostic impact of low SMI and low SMD is often reported as independent of cancer stage when using traditional clinical staging tools. Therefore, this study sought to explore the relationship between a novel marker of tumour burden (circulating tumour DNA) and skeletal muscle abnormalities at diagnosis of PDAC. A retrospective cross-sectional study was conducted in patients who had plasma and tumour tissue samples stored in the Victorian Pancreatic Cancer Biobank (VPCB) at diagnosis of PDAC, between 2015 and 2020. Circulating tumour DNA (ctDNA) of patients with G12 and G13 *KRAS* mutations was detected and quantified. Pre-treatment SMI and SMD derived from analysis of diagnostic computed tomography imaging was tested for its association to presence and concentration of ctDNA, as well as conventional staging, and demographic variables. The study included 66 patients at PDAC diagnosis; 53% female, mean age 68.7 years (SD ± 10.9). Low SMI and low SMD were present in 69.7% and 62.1% of patients, respectively. Female gender was an independent risk factor for low SMI (OR 4.38, 95% CI 1.23–15.55, *p* = 0.022), and older age an independent risk factor for low SMD (OR 1.066, 95% CI 1.002–1.135, *p* = 0.044). No association between skeletal muscle stores and concentration of ctDNA (SMI *r* = − 0.163, *p* = 0.192; SMD *r* = 0.097, *p* = 0.438) or stage of disease according to conventional clinical staging [SMI *F*(3, 62) = 0.886, *p* = 0.453; SMD *F*(3, 62) = 0.717, *p* = 0.545] was observed. These results demonstrate that low SMI and low SMD are highly prevalent at diagnosis of PDAC, and suggest they are comorbidities of cancer rather than related to the clinical stage of disease. Future studies are needed to identify the mechanisms and risk factors for low SMI and low SMD at diagnosis of PDAC to aid screening and intervention development.

## Introduction

Pancreatic ductal adenocarcinoma (PDAC) is associated with a persistently poor survival rate with only 6.7% of Australian patients surviving longer than five years from diagnosis^1^. Most patients present with advanced disease, with less than 20% of patients deemed to have resectable disease at diagnosis of PDAC^2^. Prognostication and treatment planning for PDAC hinges on thorough radiological and/or histological investigation to determine the stage and therefore resectability of disease^3,4^; this process can be complex and is recommended to be undertaken in a multidisciplinary setting^4,5^. In the majority of cases of resectable PDAC there is recurrence of disease even after surgery^6^, contributed to by the presence of subclinical metastatic disease not detectable at diagnosis using conventional staging techniques^5,7^. The degree of cancer proliferation described using clinical staging frameworks (e.g. the National Comprehensive Cancer Network (NCCN) guidelines^8^) has traditionally provided the basis for prediction of survival and planning of treatment in the context of expected outcomes^9^, due to the strong association between cancer stage and survival^10^. However, there is a need for additional biomarkers to guide clinicians in prognostication and treatment planning, and for intervention targets to improve survival outcomes in PDAC.

The measurement of cell-free DNA containing genetic mutations of a malignancy, known as circulating tumour DNA (ctDNA), is a novel biomarker of cancer burden with promising implications for improving prognostication and monitoring of disease^11^. Plasma concentration of ctDNA correlates with tumour stage and volume, and fluctuations in ctDNA levels provide a rapid and precise indication of response to systemic therapy and presence of minimal residual disease after treatment^12^. After curative therapy, detection of ctDNA is predictive of cancer recurrence^13,14^, and identifies this recurrence earlier than conventional monitoring using radiological imaging^15^. As such, ctDNA has promising utility as a biomarker in prognostication and treatment planning for PDAC.

In the setting of cancer, skeletal muscle stores are susceptible to depletion through several molecular pathways affecting metabolic, inflammatory and immune function^16–20^, although little is known about factors that initiate and regulate severity of skeletal muscle deterioration in cancer patients^18,21^. Reduction in measures of height-adjusted skeletal muscle mass (skeletal muscle index, SMI), and fatty infiltration of muscle which reduces computed tomography (CT)-assessed radiodensity (skeletal muscle radiodensity, SMD^22^) occur in addition to potential premorbid reductions precipitated by ageing, illness or injury, malnutrition, obesity, or muscle deconditioning through inactivity or degenerative disease^22–27^. Suboptimal skeletal muscle stores below sex-specific thresholds are^22^ highly prevalent in PDAC, with low SMI and low SMD reported to be present in up to 73%^28^ and 55%^29,30^ of patients, respectively.

Low SMI and low SMD are distinct phenotypical conditions which do not always coincide, conferring additive detriments to cancer outcomes^31^: low SMI is an independent prognostic indicator in PDAC^32^ and many other cancer types, associated with decreased overall survival^33–36^, reduced disease-free survival and increased risk of cancer recurrence after curative treatment^37–41^. Low SMD confers risk of reduced survival and post-operative complications in PDAC and other cancers^30,42,43^. In light of their association to important outcomes, there is interest in the potential for skeletal muscle measures (particularly SMI) to function as an additional biomarker for risk stratification and prognostication in clinical practice, and as a target for interventions in anticipation of improved outcomes^44–46^. However, the mechanism for skeletal muscle depletion influencing cancer survival is not yet clear^37,47,48^, limiting understanding of the potential impact of skeletal muscle-optimising interventions.

While significant muscle wasting has been previously associated with progression through the stages of cancer cachexia as a result of advancing disease^49^, the advent of CT-derived skeletal muscle measurement has revealed that low SMI can occur independently of weight loss or features of the cachexia syndrome^21,28,50–54^, and is also present in patients with early stages of cancer^40,55–57^. Moreover, the poor survival outcomes associated with low SMI^36^ are often independent of cancer stage, affecting patients with early, potentially curable cancers, indicating that the relationship between low SMI and reduced survival may not be a simple function of advancing or terminal disease^37,55^. The association between presence of sub-optimal skeletal muscle stores with worse survival outcomes, while having no apparent association to cancer stage in both PDAC^33,58–60^ and other cancer types^56,61–67^ has not yet been explained. For SMI, the reporting of contradictory findings in some studies^68–70^ where reduction in SMI is in fact associated with higher clinical stage of disease, complicates the interpretation of any association between these measures. There is a need to elucidate the nature of the relationship between skeletal muscle and cancer stage, to further our understanding of whether sub-optimal skeletal muscle stores are a consequence or a comorbidity of the biological process of cancer progression. This distinction has implications for the utility of skeletal muscle measurements in cancer management and treatment, through either incorporation of these measures into prognostications along with other clinical considerations, or development of interventions to optimise skeletal muscle status with the intent to improve outcomes.

Tumour interaction with the host environment contributes to skeletal muscle depletion^18^, but the effect of overall tumour burden on this wasting process, including the occult presence of micrometastases has not yet been explored. Detection and quantification of ctDNA allows for a novel method of evaluating the relationship between skeletal muscle and tumour burden, to enhance understanding about the association between skeletal muscle and disease stage, and whether overt or subclinical progression of disease contributes to the occurrence or rate of skeletal muscle deterioration. Exploration of the association between ctDNA and CT-derived measures of body composition has been previously recommended, with suggestion it may be useful in guiding prognostication and expectations around response to cancer treatment^71^, however an analysis of this kind has not yet been reported. As high recurrence rates, poor survival, and skeletal muscle depletion are characteristic of PDAC, the aim of this study was to identify risk factors for low SMI and low SMD at diagnosis, and explore the interplay between tumour burden and skeletal muscle stores. This novel investigation will enhance understanding about the association between skeletal muscle and cancer stage, and whether burden of disease contributes to deterioration in skeletal muscle stores.

## Methods

### Study design and participant selection

This study was reported according to the Strengthening of the Reporting of Observational studies in Epidemiology (STROBE) guidelines^72^. This was an exploratory retrospective cross-sectional study approved by the Monash Health Human Research Ethics Committee (Ref: RES-17-0000387L), for analysis of available data from participants of the Victorian Pancreatic Cancer Biobank (VPCB). All participants of the VPCB had provided informed consent to collection and storage of biospecimens for the purpose of future research between January 2015 and May 2020, approved by the Monash Health Human Research Ethics Committee (HREC). Criteria for inclusion in the VPCB was a diagnosis of either PDAC (all histological types), in adults over the age of 18 years. A retrospective Waiver of Consent application was approved by the Monash Health HREC, to allow for collection of additional clinical information about VPCB participants, necessary to undertake the present study. This included height and weight data, and allowed for contact with radiological imaging providers external to Monash Health in order to obtain relevant CT imaging of participants enrolled in the VPCB for whom there was an adequate plasma sample available for detection and quantification of ctDNA (> 3 mL).

Demographic and clinical information was obtained through retrospective review of medical records to determine participant gender, age, prior surgical resection, tumour location, pathological inflammatory markers (high neutrophil to lymphocyte (NLR) ratio > 3, or low albumin < 35 g/L) and clinical stage of disease according to the 2017 National Comprehensive Cancer Network (NCCN) guidelines^8^.

### Skeletal muscle analysis

Contrast-enhanced diagnostic CT imaging undertaken as part of participants’ routine clinical care, as close as possible to the date of collection of the biobank sample, was used for skeletal muscle analysis. Single-slice axial CT images at the level of the third lumbar vertebra (L3) were imported in Digital Imaging and Communications in Medicine (DICOM) format into sliceOmatic 5.0 Rev-7 software (Tomovision, Canada) for analysis using the validated technique which has been widely reported^35,73^. The range of − 29 to 150 Hounsfield Units (HU) was used to segment skeletal muscle tissue, according to the Alberta Protocol^74^. The cross-sectional skeletal muscle area was normalised for participant height to obtain the skeletal muscle index (SMI, cm^2^/m^2^). The mean radiodensity of the cross-sectional skeletal muscle area was also recorded (skeletal muscle radiodensity, SMD). Presence of skeletal muscle abnormality was determined using thresholds published by Martin et al., which was derived from a cohort of cancer patients across all categories of body mass index (BMI) and therefore congruent with the present study^35^; low SMI was defined as SMI < 41cm^2^/m^2^ in females, < 43cm^2^/m^2^ in males with a BMI ≤ 24.9 kg/m^2^, and < 53 cm^2^/m^2^ in males with a BMI > 25 kg/m^2^; in both genders, low SMD was defined as < 41HU if BMI ≤ 24.9 kg/m^2^, and < 33HU if BMI > 25 kg/m^2^.

### Circulating tumour DNA analysis

Samples of frozen plasma and tumour tissue taken from diagnostic biopsy or surgical resection were retrieved from the Victorian Pancreatic Cancer Biobank. Mutational status of tumour tissue samples was detected using a *KRAS* StripAssay (ViennaLab Diagnostics, Vienna, Austria). Samples with *KRAS* mutations on codons 12 and 13 were included in the analysis. ctDNA analysis was conducted using the *KRAS* G12/G13 Screening Kit (Bio-Rad, Hercules, CA, USA) according to the manufacturer’s recommended protocols. G12/G13 *KRAS* mutations were detected via droplet digital PCR (ddPCR), with inclusion of positive and negative controls, and all samples were analysed in duplicate. Detection and quantification of ctDNA was conducted using QuantaSoft software (Bio-Rad, Hercules, CA, USA), with the lower limit of detection for a positive ctDNA result set at three or more droplets^75^. The mutant allele fraction (MAF) represents the proportion of mutant to total allele detected.

### Statistical analysis

Data were tested for normality using the Shapiro–Wilk test. Differences between groups stratified by SMI or SMD status were evaluated using independent samples t-tests or Mann–Whitney U tests for continuous variables. The chi-squared test for independence or Fishers-Exact test were used to investigate difference between groups for categorical variables. Pearson’s correlation test was used for analysis of relationships between most continuous variables; Spearman’s rank correlation was used for non-parametric data (concentration of ctDNA).

Differences in SMI, SMD, and concentration of ctDNA between clinical stages of PDAC were examined using the Kruskal–Wallis test or one-way analysis of variance. The Jonckheere–Terpstra test for ordered alternatives was used to investigate the trend of median ctDNA concentration across advancing clinical stages of PDAC. Concentration of ctDNA was median dichotomised for comparison between participants with low or undetected ctDNA vs those with higher levels of ctDNA.

Univariate logistic regression was used to determine odds ratios and 95% CIs to test for variables associated with low SMI and low SMD at diagnosis of PDAC. For this exploratory study, variables with a significance of *p* < 0.25 on univariate analysis were included in a multiple logistic regression model^76^. Analyses of differences in SMI and SMD between genders and between clinical stages was additionally conducted in a larger sample comprising participants with available data who had been initially excluded due to type of mutation or lack of plasma for ctDNA analysis, for comparison with the primary study cohort. Statistical analyses were conducted using SPSS software (Version 27). All statistical tests were two-sided, with a *p* value of < 0.05 indicating statistical significance.

### Ethics approval and consent to participate

This research was conducted in accordance with the principles of the 1964 Declaration of Helsinki and its later amendments. All participants of the VPCB provided informed consent to collection and storage of biospecimens for the purpose of future research between January 2015 and May 2020, approved by the Monash Health HREC (Ref: 15450A). A retrospective Waiver of Consent application was approved by the Monash Health HREC (HREC/15/MonH/117), to allow for collection of additional clinical information about VPCB participants, necessary to undertake the present study.

## Results

### Participant selection

**Figure 1:**
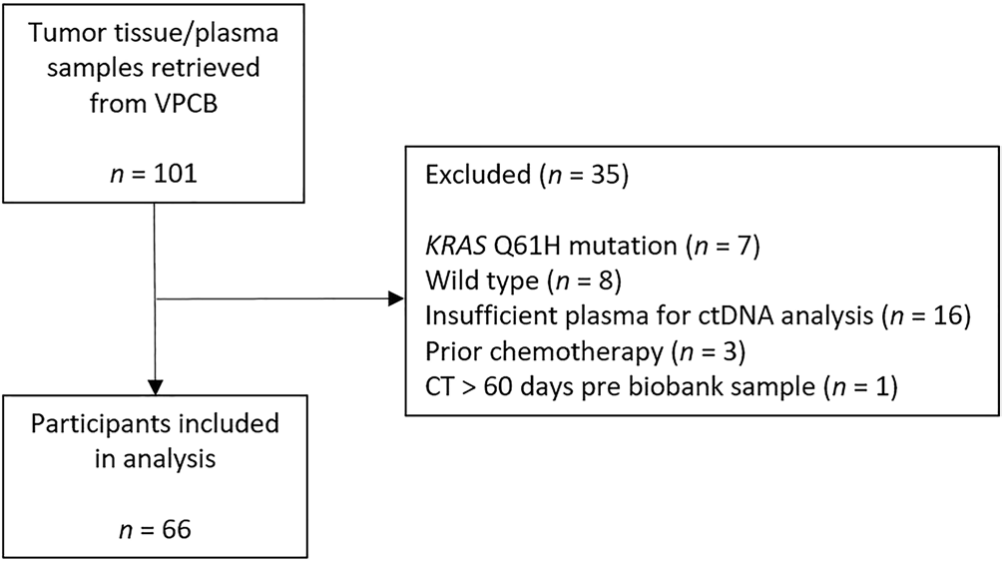
Flowchart of participant selection for the present analysis. *VPCB* Victorian Pancreatic Cancer Biobank, *KRAS* Kirsten rat sarcoma viral oncogene homolog, *ctDNA* circulating tumour DNA, *CT* computed tomography.

The flowchart for selection of participants is presented in Fig. 1. One-hundred-and-one participants were identified as having both pancreatic tumour tissue and plasma samples stored within the VPCB (‘the biobank’). Tissue samples for these participants were retrieved from the biobank and tested for *KRAS* oncogene mutations. Eighty-six samples were positive for G12/13 *KRAS* mutations, and 15 were either wild type or harboured Q61H mutations and were therefore excluded from the analysis. ctDNA could not be quantified for 16 participants due to insufficient plasma in the biobank sample. Participants who had commenced chemotherapy prior to CT imaging or biobank sampling (*n* = 3) were excluded, to remove the confounding effect of chemotherapy on both measures of skeletal muscle and concentration of ctDNA^12,77^. Participants who had undergone CT imaging > 60 days prior to biobank sampling (*n* = 1) were also excluded from the analysis. The median time difference between CT imaging and ctDNA sampling was 6 days pre-biopsy (IQR 15 days pre, to 2 days post).

### Participant characteristics

Demographics and clinical characteristics of all study participants (*n* = 66) are presented in Table 1. The mean participant age was 68.7 (± 10.9) years, and 53% of participants were female. Most participants were diagnosed with pancreatic head and/or neck tumours, and systemic inflammation (NLR ≥ 3 or albumin < 35 g/L) was present in around half of participants at diagnosis.

**Table 1:** Participant characteristics at diagnosis of PDAC.

	All (*n* = 66)	Males (*n* = 31)	Females (*n* = 35)	*p*
Demographics				
Age (years)	68.7 (10.9)	67.8 (12.9)	69.5 (9.0)	0.529
BMI (kg/m^2^)	25.8 (4.8)	25.4 (3.9)	26.1 (5.5)	0.544
Clinical characteristics				
Tumour location, n (%)				
Head and neck	52 (78.8)	26 (83.9)	26 (74.3)	0.672
Body and tail	12 (18.2)	8 (25.8)	4 (11.4)	
Head/neck and body	2 (3.0)	1 (3.20)	1 (2.9)	
Clinical stage, n (%)				
Resectable	23 (34.8)	10 (32.2)	13 (37.1)	0.867
Borderline resectable	9 (13.6)	5 (16.1)	4 (11.4)	
Locally advanced	13 (19.7)	7 (22.6)	6 (17.1)	
Metastatic	21 (31.8)	9 (29.0)	12 (34.3)	
NLR, n (%)^a^				
Normal (≤ 3)	28 (42.4)	14 (48.3)	14 (45.2)	1.000
High (> 3)	32 (48.5)	15 (51.7)	17 (54.9)	
Albumin, n (%)^b^				
Normal (≥ 35 g/L)	23 (34.8)	9 (33.3)	14 (46.7)	0.451
Low (< 35 g/L)	34 (51.5)	18 (66.7)	16 (53.3)	
ctDNA detection, n (%)				
Negative	17 (25.8)	10 (32.3)	7 (20.0)	0.393
Positive	49 (74.2)	21 (67.7)	28 (80.0)	
Concentration of ctDNA (MAF)^c^	0.126 (0–0.699)	0.098 (0–0.269)	0.162 (0.038–0.703)	0.294
High/low ctDNA, n (%)^d^				
Negative/low	33 (50)	17 (54.8)	16 (45.7)	0.622
High	33 (50)	14 (45.2)	19 (54.3)	
Skeletal muscle analysis				
SMI (cm^2^/m^2^)	40.9 (7.6)	45.9 (7.3)	36.4 (4.4)	< 0.001
SMD (mean HU)	34.0 (11.2)	35.8 (10.5)	32.4 (11.6)	0.212
Low SMI, n (%)	46 (69.7)	16 (53.3)	30 (85.7)	0.006
Low SMD, n (%)	41 (62.1)	18 (58.1)	23 (65.7)	0.700

Continuous data presented as mean (standard deviation) unless otherwise indicated. *BMI* body mass index, *CT* computed tomography, *ctDNA* circulating tumour DNA, *MAF* mutant allele fraction, *NLR* neutrophil to lymphocyte ratio, *PDAC* pancreatic ductal adenocarcinoma, *SMD* skeletal muscle radiodensity, *SMI* skeletal muscle index; ^a^missing data for 6 participants (n = 60); ^b^missing data for 9 participants (n = 57); ^c^data presented as median (interquartile range); ^d^median dichotomised.

**Figure 2:**
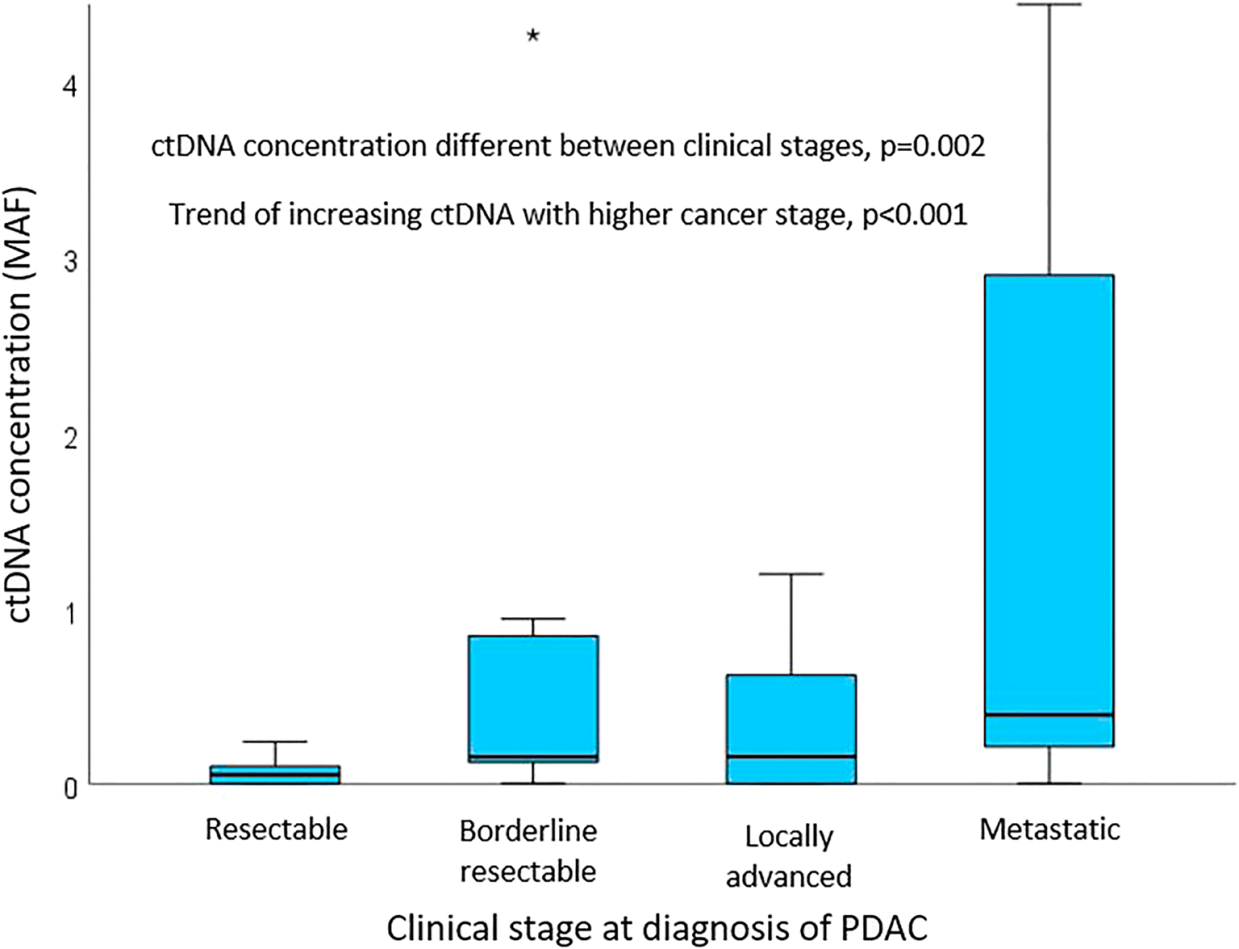
Boxplot of concentration of circulating tumour DNA (ctDNA) of participants grouped by clinical stage at diagnosis of pancreatic ductal adenocarcinoma (PDAC), *n* = 66. *MAF* mutant allele fractions; outliers included in analysis but not shown in graph, to enable visual representation.

Fifty-two percent of participants had unresectable tumours at diagnosis; almost a third of participants had metastatic disease. ctDNA was detected in 74% of participants (Table 1). Concentration of ctDNA was significantly different between stages of disease [*H*(3) = 15.307, *p* = 0.002], with a significant trend of increasing ctDNA concentration associated with more advanced disease at diagnosis defined by NCCN clinical stage (*J* = 1103.5, *z* = 3.737, *p* < 0.001, *r* = 0.46) (Fig. 2). There was no correlation between concentration of ctDNA and age (*r* = 0.090, *p* = 0.470) or BMI (*r* = − 0.107, *p* = 0.393).

Factors associated with skeletal muscle stores at diagnosis of pancreatic ductal adenocarcinoma.

### Skeletal muscle index

**Figure 3:**
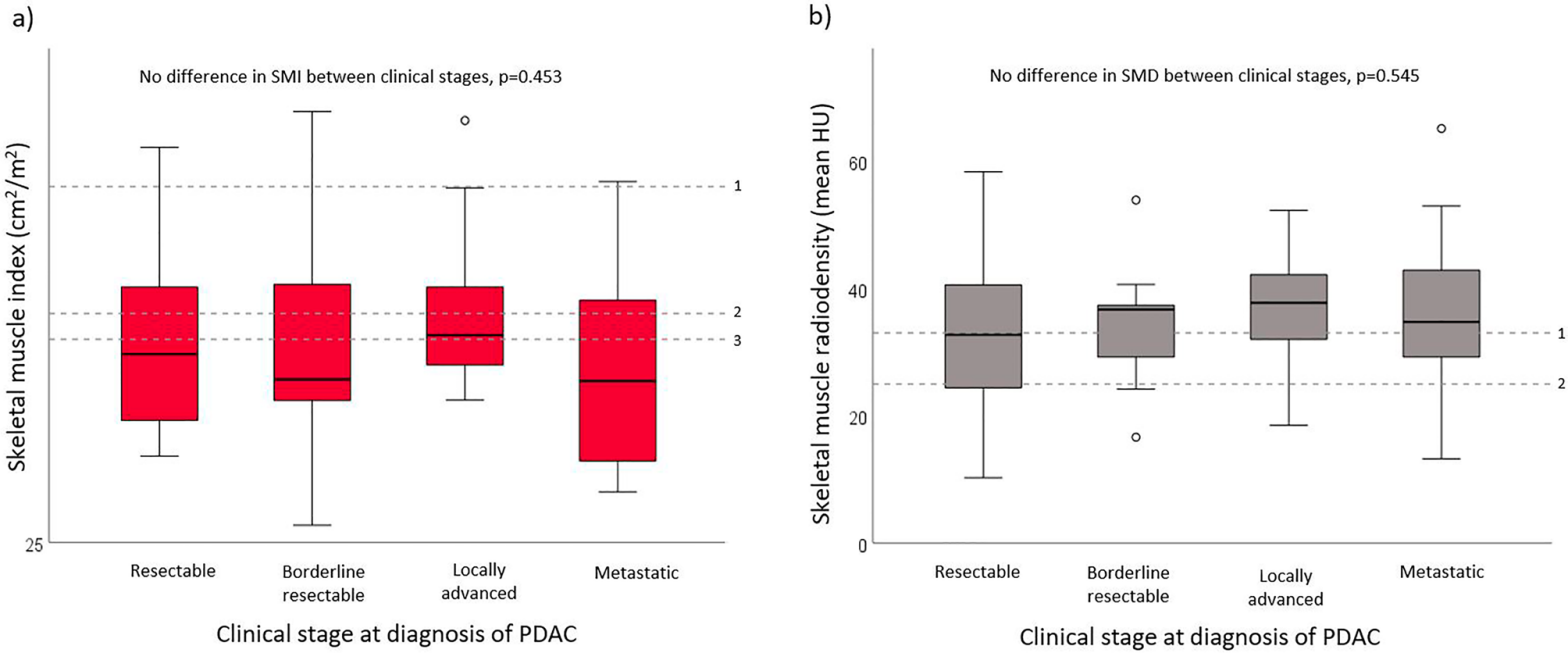
Boxplots of participants’ skeletal muscle stores grouped by clinical stage at diagnosis of pancreatic ductal adenocarcinoma (PDAC), n = 66. Reference lines show thresholds for low SMI/SMD according to Martin et al.^35^. (**a**) Skeletal muscle index (SMI), no difference between clinical stages; (1) males with BMI ≥ 25 kg/m^2^; (2) males with BMI < 25 kg/m^2^; (3) females (all). (**b**) Skeletal muscle radiodensity (SMD), no difference between clinical stages; (1) BMI < 25 kg/m^2^; (2) BMI ≥ 25 kg/m^2^; *HU* Hounsfield Units.

The prevalence of low SMI was 69.7% for the whole group. Females had a lower mean SMI than males (*p* < 0.001), and a significantly higher prevalence of low SMI than males (85.7% vs. 53.3%, *p* = 0.006) (Table 1). There was a weak, negative correlation between SMI and age (*r* = − 0.245, *p* = 0.047), but no significant correlation with BMI (*r* = − 0.191, *p* = 0.125). There was no statistical difference in SMI between clinical stages of PDAC at diagnosis (*F*(3, 62) = 0.886, *p* = 0.453) (Fig. 3a), and no significant linear correlation between concentration of ctDNA and SMI (*r* = − 0.163, *p* = 0.192). In a multiple regression analysis, female gender was independently associated with higher odds of having low SMI, after controlling for increasing age and high NLR (Table 2).

**Table 2:** Association between clinicopathological characteristics of participants, and SMI status (low vs normal), at diagnosis of PDAC.

	Low SMI (*n* = 46)	Normal SMI (*n* = 20)	*p*	Univariate analysis OR (95% CI)	*p*	Multivariable analysis OR (95% CI)	*p*
Age (years)	70.8 (9.0)	63.8 (13.4)	0.039	1.064 (1.010–1.121)	0.020	1.040 (0.976–1.107)	0.225
Gender, n (%)							
Male	16 (34.8)	15 (75)	0.006	1			
Female	30 (65.2)	5 (25)		5.625 (1.728–18.307)	0.004	4.380 (1.234–15.547)	0.022
BMI (kg/m^2^)	26.1 (5.2)	25.0 (3.9)	0.390	1.052 (0.938–1.181)	0.386		
Tumour location, n (%)							
Body and tail	9 (19.6)	3 (15)	0.743	1			
Head and neck ± body	37 (80.4)	17 (85)		0.725 (0.174–3.023)	0.659		
Clinical stage, n (%)							
Resectable	17 (37.0)	6 (30)	0.784	1			
Borderline resectable	5 (10.9)	4 (20)		0.441 (0.088–2.209)	0.319		
Locally advanced	9 (19.6)	4 (20)		0.794 (0.177–3.563)	0.763		
Metastatic	15 (32.6)	6 (30)		0.882 (0.234–3.328)	0.853		
Resectability							
Potentially resectable	22 (47.8)	10 (50)	1.0	1			
Unresectable	24 (52.2)	10 (50)		1.091 (0.382–3.118)	0.871		
NLR, n (%)^a^							
Normal (≤ 3)	16 (34.8)	12 (60)	0.080	1			
High (> 3)	26 (56.5)	6 (30)		3.250 (1.018–10.379)	0.047	2.445 (0.624–9.576)	0.199
Albumin, n (%)^b^							
Normal (≥ 35 g/L)	17 (37.0)	6 (30)	1.0	1			
Low (< 35 g/L)	24 (52.2)	10 (50)		0.847 (0.258–2.778)	0.784		
ctDNA detection, n (%)							
Negative	10 (21.7)	7 (35.0)	0.409	1			
Positive	36 (78.3)	13 (65.0)		1.938 (0.610–6.156)	0.262		
Concentration of ctDNA (MAF)^c^	0.126 (0.019–0.345)	0.125 (0–1.108)	0.944	0.997 (0.866–1.148)	0.971		
High/low ctDNA, n (%)^d^							
Negative/low	23 (50)	10 (50)	1.000	1			
High	23 (50)	10 (50)		1.000 (0.350–2.857)	1.000		

Continuous data presented as mean (standard deviation) unless otherwise indicated; *BMI* body mass index, *CT* computed tomography, *ctDNA* circulating tumour DNA, *MAF* mutant allele fraction, *NLR* neutrophil to lymphocyte ratio, *PDAC* pancreatic ductal adenocarcinoma; ^a^missing data for 6 participants, *n* = 60; ^b^missing data for 9 participants, *n* = 57; ^c^data presented as median (interquartile range); ^d^median dichotomised.

A comparative analysis was conducted in a larger sample of participants (*n *= 89) which included 23 additional participants who were previously excluded due to type of mutation or lack of plasma for ctDNA analysis [48% female, mean age 69.0 (± 10.8) years]. As observed in the smaller primary cohort (*n* = 66), low SMI was more highly prevalent in females (84% vs 54%,* p* = 0.006) and there was no difference in SMI between clinical stages of PDAC at diagnosis (*F*(3, 85) = 0.580, *p* = 0.630, *n* = 89).

### Skeletal muscle radiodensity

Low SMD was present in 62.1% of participants, with no gender differences in prevalence (Table 1). There was a moderate, negative correlation between SMD and age (*r* = − 0.415, *p* < 0.001) and BMI (*r* = − 0.491, *p* < 0.001). There was no statistical difference in SMD between clinical stages of PDAC at diagnosis (*F*(3, 62) = 0.717, *p* = 0.545), and no significant linear correlation between concentration of ctDNA and SMD (*r* = 0.097, *p* = 0.438). In a multivariable analysis, increasing age remained independently associated with increased odds of having low SMD after controlling for unresectable disease, BMI, high NLR, and concentration of ctDNA (Table 3). In the comparative analysis using a larger cohort (*n* = 89), similar results were observed: there was no difference in prevalence of low SMD between genders (male 50%, female 65%, *p* = 0.220), and no difference in SMD between clinical stages of PDAC at diagnosis (*F*(3, 85) = 1.314, *p* = 0.275).

**Table 3:** Association between clinicopathological characteristics of participants, and SMD status (low vs normal), at diagnosis of PDAC.

	Low SMD (*n* = 41)	Normal SMD (*n* = 25)	*p*	Univariate analysis OR (95% CI)	*p*	Multivariable analysis OR (95% CI)	*p*
Age (years)	71.4 (9.0)	64.3 (12.5)	0.010	1.066 (1.013–1.121)	0.014	1.066 (1.002–1.135)	0.044
Gender, n (%)							
Male	18 (43.9)	13 (52.0)	0.700	1			
Female	23 (56.1)	12 (48.0)		1.384 (0.51–3.755)	0.523		
BMI (kg/m2)	26.5 (5.1)	24.7 (4.2)	0.153	1.085 (0.969–1.215)	0.156	1.030 (0.909–1.167)	0.645
Tumour location, n (%)							
Body and tail	8 (19.5)	4 (16.0)	1.000	1			
Head and neck ± body	33 (80.5)	21 (84.0)		0.786 (0.210–2.938)	0.720		
Clinical stage, n (%)							
Resectable	17 (41.5)	6 (24.0)	0.393	1			
Borderline resectable	6 (14.6)	3 (12.0)		0.706 (0.133–3.748)	0.683		
Locally advanced	6 (14.6)	7 (28.0)		0.303 (0.072–1.269)	0.102^c^		
Metastatic	12 (29.3)	9 (36.0)		0.471 (0.132–1.676)	0.245		
Resectability							
Potentially resectable	23 (56.1)	9 (36.0)	0.183	1			
Unresectable	18 (43.9)	16 (64.0)		0.440 (0.158–12..5)	0.116	0.547 (0.160–1.867)	0.335
NLR, n (%)^a^							
Normal (≤ 3)	15 (36.6)	13 (52.0)	0.347	1			
High (> 3)	22 (53.7)	10 (40.0)		1.907 (0.665–5.469)	0.230	1.587 (0.450–5.590)	0.472
Albumin, n (%)^b^							
Normal (≥ 35 g/L)	14 (34.1)	9 (36.0)	0.808	1			
Low (< 35 g/L)	23 (56.1)	11 (44.0)		1.344 (0.446–4.052)	0.599		
ctDNA detection, n (%)							
Negative	10 (24.4)	7 (28.0)	0.972	1			
Positive	31 (75.6)	18 (72.0)		1.206 (0.391–3.721)	0.745		
Concentration of ctDNA (MAF)^d^	0.010 (0.008–0.361)	0.154 (0–0.957)	0.390	0.865 (0.733–1.021)	0.086	0.860 (0.725–1.019)	0.082
High/low ctDNA, n (%)^e^							
Negative/low	22 (53.7)	11 (44.0)	0.612	1			
High	29 (46.3)	14 (56.0)		0.679 (0.250–1.845)	0.447		

Continuous data presented as mean (standard deviation) unless otherwise indicated; *BMI* body mass index, *CT* computed tomography, *ctDNA* circulating tumour DNA, *MAF* mutant allele fraction, *NLR* neutrophil to lymphocyte ratio, *PDAC* pancreatic ductal adenocarcinoma; ^a^missing data for 6 participants, *n* = 60: ^b^missing data for 9 participants, *n* = 57; ^c^Locally advanced stage not added to multivariable model individually as ‘unresectable’ group was entered (*p* < 0.25); ^d^data presented as median (interquartile range); ^e^median dichotomised.

## Discussion

This study aimed to explore the association between skeletal muscle stores and tumour burden (using ctDNA as a novel biomarker as well as traditional clinical staging) at diagnosis of PDAC. Skeletal muscle abnormalities were found to be highly prevalent at diagnosis of PDAC in this cohort (low SMI 69.7%, low SMD 62.1%). Presence of low SMI or low SMD was not associated with clinical stages of PDAC, and neither presence nor concentration of ctDNA was associated with increased likelihood of having low SMI or SMD at diagnosis of PDAC. Instead, female gender was the strongest predictor of low SMI, and increasing age was associated with low SMD. These findings suggest that the deterioration of skeletal muscles stores in people diagnosed with PDAC may occur early in the disease process, and are not simply a direct result of advancing clinical stage or increasing tumour burden.

To better understand the association between tumour burden and skeletal muscle status we studied circulating tumour DNA, which is a more sensitive marker of cancer progression than standard clinical staging measures^15^. The analysis of this relationship is novel, and has not been conducted in any cancer type. In this exploratory study, concentration of ctDNA increased with progressively higher clinical stage of disease as expected^78,79^, but there was no association between detection or concentration of ctDNA, with SMI or SMD measures in this cohort of patients with PDAC. The significant increase in ctDNA concentration with higher cancer stage, while having no association to skeletal muscle measures, supports the findings from other studies that there is no clear association between sub-optimal skeletal muscle stores and clinical stage of disease^57,59^.

As low skeletal muscle stores are prevalent comorbidities of PDAC, it is important to understand the factors that are associated with their presence to enable identification and intervention. Nearly three in four participants had low SMI at diagnosis, which is consistent with other reports^28,53^. The lack of difference in BMI between participants with low or normal SMI reinforces the notion that basic anthropometric methods cannot be relied upon to distinguish those at risk of skeletal muscle depletion, which can occur independent of change in body weight^53,80,81^. Presence of low SMI was strongly associated with female gender in this study, but this remains contentious in the PDAC literature where most analyses are univariate; some studies report a similar finding^82,83^, however in other studies no gender differences are seen^42,84^. Classification of gender as a risk factor for low SMI has potential importance in screening of PDAC patients to identify those most vulnerable to skeletal muscle depletion, but there is a dearth of multivariate investigations of factors influencing SMI in PDAC patients. In the single study where factors influencing SMI prior to resection of PDAC have been investigated using multivariate regression analysis (*n* = 273), the association with female gender aligned with our findings^53^. Future multivariate analyses in larger cohorts are needed to confirm our finding that female gender is a risk factor for low SMI in PDAC.

Low SMD is more prevalent in PDAC than most other cancers, though the reason for this is not known^85^. In our study, low SMD was identified in 62% of participants which is a higher prevalence than has been previously reported^29,30,85,86^. Increasing age was associated with higher likelihood of having low SMD in our cohort, which aligns with results of studies in other cancer types^21,51^; to our knowledge, this is the first study to explore risk factors for low SMD in PDAC patients. Ageing is both a risk factor for development of cancer^87^ and associated with the characteristics of obesity, diabetes and inactivity which are also linked to reduction in SMD^22,23^, which may explain this association. Low SMD has also been associated with the presence of inflammatory markers such as high NLR or low albumin level^21^, however the evidence-base is limited. This association was not demonstrated in the present study but this may be attributed to the small sample size. Similar to SMI, there are several studies reporting a lack of association between low SMD and clinical stage of disease^42,56,67^, indicating that low SMD is a comorbidity of cancer with a potentially complex aetiology involving demographic, phenotypic, and lifestyle characteristics^22^. While the aetiology of intramuscular lipid accumulation resulting in reduced skeletal muscle radiodensity in the cancer population is poorly understood, it has been identified that both history of cigarette smoking and presence of comorbidities such as diabetes are associated with low SMD^21,88^. These characteristics are also risk factors for developing PDAC^89^, which may explain why prevalence of low SMD in PDAC is high.

The focus on a single cancer type, with participants studied prior to commencement of treatment, is a strength of this study. Additionally, a single, trained investigator conducted all CT-derived skeletal muscle analyses and as a result, the main between-participant differences in variables included in the analysis were natural variations in stage or location of disease, and phenotypical characteristics such as age and BMI. Limitations of this exploratory study are its small sample size and convenience sampling approach which is acknowledged to affect the generalisability of results. Moreover, the retrospective design precluded statistical adjustment for factors known to have an association with SMI or SMD such as nutrition status and/or weight loss history^46^, presence of comorbidities^88^, smoking history^21^, strength or physical activity level^90,91^, or impairment of digestion due to pancreatic enzyme insufficiency^92^. These factors should be incorporated into future prospective study designs with larger sample sizes to enhance external validity.

The findings of the present study suggest that low SMI and low SMD are comorbidities of PDAC. Our data suggest that women with PDAC are more likely to have low SMI, and those with older age more likely to have low SMD at diagnosis of PDAC. Screening is needed to identify sub-optimal skeletal muscle status in people with PDAC who are at greatest risk of poor outcomes, because it is not easily identified by indices such as BMI. Future research is required to further elucidate potential mechanisms and risk factors for low SMI and low SMD at diagnosis of PDAC; the lack of association between ctDNA, a novel, sensitive marker of disease burden, and SMI or SMD, suggests that sub-optimal skeletal muscle conditions are not simply a function of advancing cancer stage. Assessment of skeletal muscle stores in clinical practice therefore has utility beyond prognostication, and therapy for people with PDAC should also consider interventions aimed at improving muscle status alongside treatment for the underlying disease.

## Data Availability

The ethical approval obtained to conduct this study does not extend to sharing of data, therefore the data supporting the findings of this study are not openly available. De-identified data may be shared upon reasonable request to the corresponding author if proposed research has undergone ethical review.
